# Targeting macrophage anti-tumor activity to suppress melanoma progression

**DOI:** 10.18632/oncotarget.14474

**Published:** 2017-01-03

**Authors:** Huafeng Wang, Lijuan Zhang, Luhong Yang, Chengfang Liu, Qi Zhang, Linjing Zhang

**Affiliations:** ^1^ Modern College of Arts and Science, or School of Life Science, Shanxi Normal University, Linfen, China; ^2^ Research Center of Basic Medical Sciences, Tianjin Medical University, Tianjin, China; ^3^ Department of Human Anatomy, Shanxi Medical University, Shanxi Sheng, China; ^4^ Nankai Hospital, Tianjin, China

**Keywords:** melanoma, macrophage, GM-CSF, immunoadjuvant agent

## Abstract

By phagocytosing cancer cells and their cellular debris, macrophages play a critical role in nonspecific defense (innate immunity) and, as antigen presenters, they help initiate specific defense mechanisms (adaptive immunity). Malignant melanoma is a lethal disease due to its aggressive capacity for metastasis and resistance to therapy. For decades, considerable effort has gone into development of an effective immunotherapy for treatment of metastatic melanoma. In this review, we focus on the anti-tumor activities of macrophages in melanoma and their potential as therapeutic targets in melanoma. Although macrophages can be re-educated through intercellular signaling to promote tumor survival owing to their plasticity, we expect that targeting the anti-tumor activity of macrophages remains a promising strategy for melanoma inhibition. The combination of tumoricidal macrophage activation and other treatments such as surgery, chemotherapy, and radiotherapy, may provide an effective and comprehensive anti-melanoma strategy.

## INTRODUCTION

Melanoma is one of the most dangerous cancers, illustrated by the fact that it represents less than 5% of all skin cancers but results in the majority of skin cancer-related deaths [1]. In 2012, 232,000 people were diagnosed with melanoma globally and the disease resulted in 55,000 deaths [2]. It is estimated that in the United States there were 73,870 new cases and 9,940 deaths from melanoma in 2015 [3]. In mainland China, there are estimated to be about 20,000 new cases of melanoma annually [4], among which about 50-70% are primary skin melanoma skin and 6% are melanoma of the mucous membrane [5]. Malignant melanoma is lethal because of its aggressive capacity for metastasis and resistance to therapy [1]. Tumors can potentially be recognized as “altered self,” akin to allogeneic immunity, leading to an anti-tumor immune response of potential value in the adjuvant setting. Therefore, researchers have begun to develop immunotherapy for treatment of metastatic melanoma [6]. This has motivated investigation of interactions between melanoma and immune cells and translation of this knowledge into effective clinical strategies.

Macrophages, first discovered by Elie Metchnikoff in 1884 [7], are a type of white blood cell, and also prodigious and industrious “janitors” in our body that engulf and digest junk or malignant cells, such as cancer cells or cellular debris in a process called phagocytosis [8]. Besides phagocytosis, they play a critical role in nonspecific defense (innate immunity) and, in their role as antigen presenters help initiate specific defense mechanisms (adaptive immunity) by recruiting other immune cells such as lymphocytes.

Macrophages, an important component of the innate immunity against tumors, are attracted by locally secreted chemokines [9]. Activated macrophages defend against tumors by direct tumor cytotoxicity and by secreting cytokines to recruit secondary immune cells, presenting antigen to T cells [10, 11], including melanoma [12, 13]. However, owing to their plasticity, macrophages can be re-educated to alter their phenotype [14, 15]. Substantial evidence indicates that macrophages in the context of the tumor microenvironment, rather than being tumoricidal, adopt a pro-tumor phenotype *in vivo* both in the primary and metastatic sites by the presence of growth factors in the tumor microenvironment as well as by intercellular interactions [14, 16]. Consequently, there is a duality in the function of macrophages in tumor.

In this review, we focus on the anti-tumor role of macrophages in melanoma and its potential for therapeutic targeting.

## ADOPTIVE TRANSFER OF ACTIVATED *IN VITRO* MACROPHAGES

Cytotoxic macrophages may occupy a major role in the defense mechanism to neoplasia [17]. While normal macrophages do not appear to be effective in attacking tumors, they can be induced to be cytotoxic *in vitro* by application of specific activators and then adoptive transfer can be performed on these induced macrophages to bring forth their anti-tumor effects *in vivo*.

### Xenogeneic macrophage activation strategies

**Figure 1:**
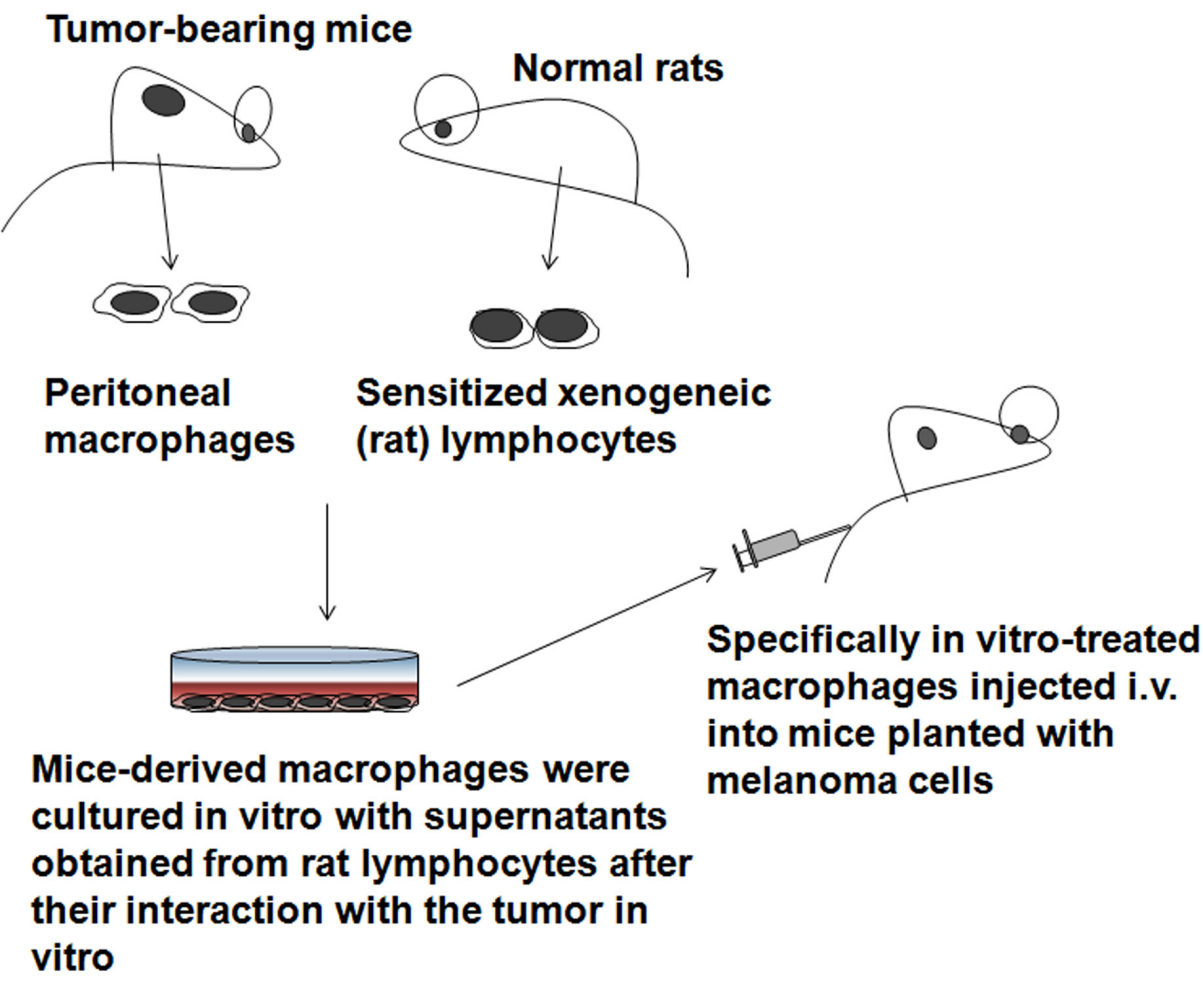
Xenogeneic activation of macrophages strategies These macrophages were cultured *in vitro* with various supernatants obtained from xenogeneic lymphocytes after their interaction with the tumor *in vitro*

Macrophages can be induced to be cytotoxic *in vitro* using supernatants obtained from sensitized xenogeneic lymphocytes (Figure 1). The application of xenogeneic activated macrophages from tumor-bearing animals rendering them cytotoxic may provide a possible approach to therapy. As early as 1974, investigators studied the ability of syngeneic macrophages from C57BL/6 mice bearing a progressively growing B16 melanoma to inhibit established pulmonary metastases *in vivo* [12]. Peritoneal macrophages were isolated from C57BL/6 mice bearing progressively growing subcutaneous B16 melanoma that had been treated with thioglycollate. The macrophages were cultured *in vitro* with supernatants obtained from xenogeneic lymphocytes after their interaction with the tumor *in vitro* and were injected intravenously (i.v.) into other C57BL/6 mice that had been given i.v. injections of 10,000 viable B16 melanoma cells 48 hr previously. *In vitro*-treated macrophages injected i.v. into mice significantly reduced their number of established pulmonary metastases. Moreover, it appeared that the *in vivo* inhibition of tumor nodules was continuing at the time of sacrifice [12].

### Stimulating factor strategies

Stimulated lymphocytes release a large number of biologically active mediators, some of which are chemotactic to macrophages, activating the macrophages and rendering them cytotoxic [17]. The potential anti-tumor activity of human macrophages, grown in macrophage colony stimulating factor (M-CSF), was examined in mice homozygous for the severe combined immune deficiency (SCID) mutation, bearing xenografts of autologous human melanoma [18]. Injection of the cultured macrophages, once or repeatedly, resulted in partial to complete regression of tumors [18].

### Microbe-associated factors

Muramyl dipeptide (MDP) is the active immunomodulating component of mycobacterial cell walls contained in Freund's complete adjuvant [19]. Regardless of whether they are alveolar, peritoneal or hepatic, *in vitro* exposure of macrophages to MDP has been shown to render these cells tumoricidal in melanoma. F344 rat alveolar macrophages can be rendered tumoricidal against xenogeneic melanoma cells following incubation with both unencapsulated (free) MDP and liposome-encapsulated MDP [19]. Cultured liver macrophages can also be activated *in vitro* with both unencapsulated (free) MDP and liposome-encapsulated MDP to a tumoricidal state against melanoma [20]. Moreover, it was observed that encapsulation of MDP within liposomes substantially augments the MDP-induced cytotoxicity [20].

## ACTIVATING MACROPHAGES *IN VIVO* TO FIGHT AGAINST MELANOMA

Cytotoxic macrophages against syngeneic tumor cells can also be induced *in vivo*, for example, by injecting immunomodulator-loaded liposomes intravenously [21].

### GM-CSF

Among stimulating factors, GM-CSF is widely used to stimulate macrophages to become tumoricidal, and it requires no additional factors [22]. As a potential approach to adjuvant immunotherapy, sequential intralesional administration of GM-CSF in dermal and subdermal melanoma lesions can induce immense antitumor immune response [23]. Evidence has been accumulated that GM-CSF is useful as an immunoadjuvant agent for cancer vaccines [24]. Hogge and colleagues injected healthy dogs with lethally irradiated canine melanoma cells transfected with human GM-CSF and saw increased numbers of macrophages at the vaccination site [25]. In a further trial, Finocchiaro and Gilkin combined autologous/allogeneic formolized tumor cells as a vaccine injected concomitantly with lethally irradiated xenogeneic cells producing human GM-CSF to treat canine malignant melanoma patients [26]. In another surgery adjuvant approach, GM-CSF was combined with the periodic administration of lipoplexes carrying the gene of human GM-CSF at the time of surgery [27]. It was demonstrated that this surgery adjuvant combined treatment was significantly delayed or prevented postsurgical recurrence and distant metastasis, increasing disease-free and overall survival, and maintaining the quality of life [27].

### Immunoembolization

**Figure 2:**
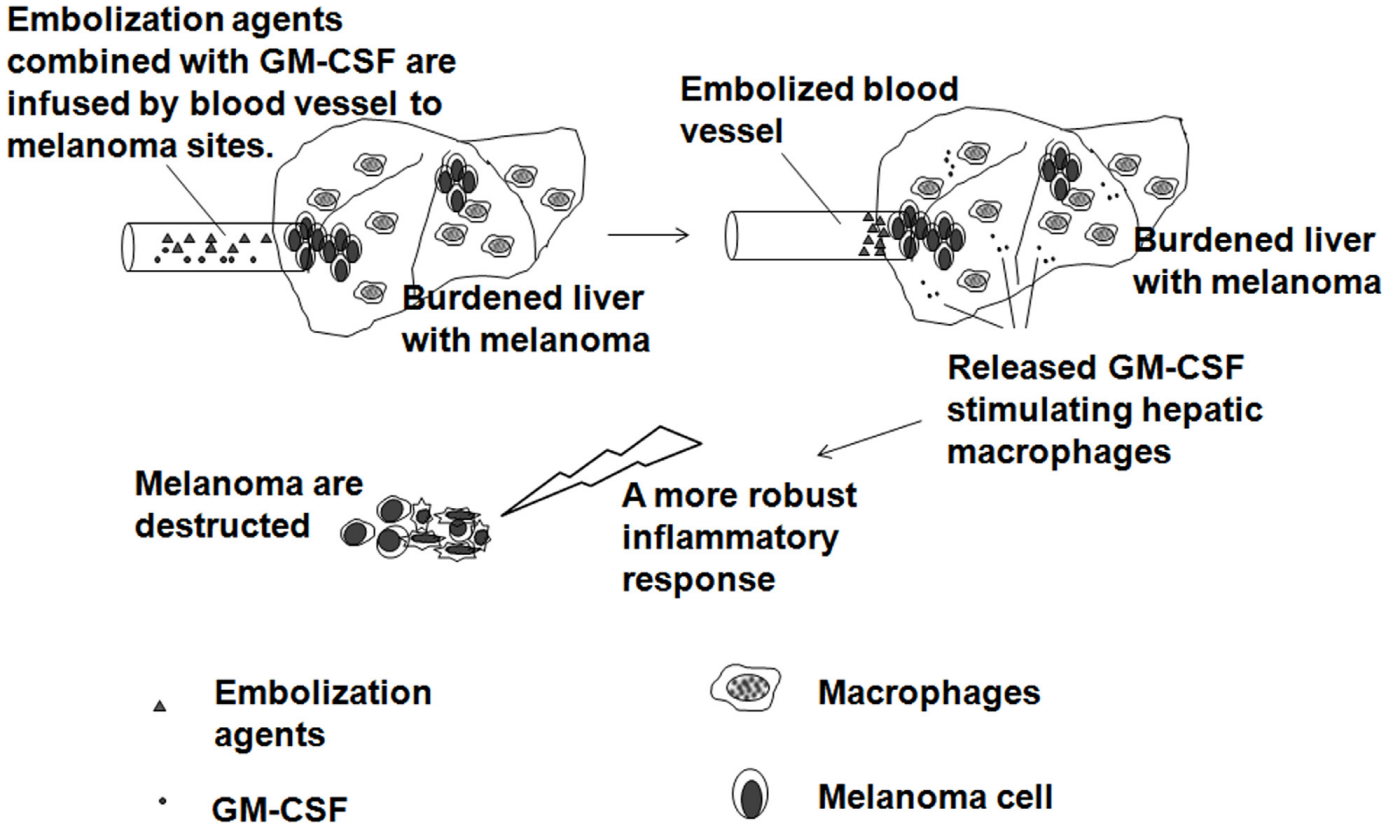
Immunoembolization with tumoricidal macrophage aggregation Embolization agents combined with GM-CSF are infused by blood vessel to melanoma sites, which results in disruption of the tumor blood supply and the local immunologic reaction evoked by GM-CSF stimulating hepatic macrophages.

GM-CSF, as an immunoadjuvant agent, has been used for the embolization of human melanoma. In 2008, a phase I trial that used human recombinant GM-CSF for immuno- embolization was reported [28]. There was no maximum-tolerated, dose-limiting dose or late toxicity found at doses as high as 2000 μg of GM-CSF, and higher doses correlated with longer systemic progression-free survival. A subsequent retrospective analysis that compared immuno- embolization with carmustine chemoembolization showed a significantly longer survival with immuno- embolization [29]. To further investigate the immunologic mechanism and efficacy of this approach, a randomized phase II clinical trial was designed. Immunoembolization with GM-CSF mixed with ethiodized oil was performed on patients with histologically confirmed metastatic uveal melanoma to the liver (contains more than 70% of all tissue macrophages) [30]. The investigators expected that local immunologic reaction evoked by GM-CSF would induce systemic immunity against melanoma cells and delay the development of remote systemic metastases [30]. The working hypothesis of the study was that immuno- embolization would stimulate massive numbers of hepatic macrophages to induce a more robust inflammatory response, triggering a systemic immune recognition of uveal melanoma and delaying the progression of extrahepatic metastasis (Figure 2). These patients showed longer extrahepatic recurrence-free periods, presumably as a result of better immunologic control of circulating micrometastases [30]. The prognosis of patients with uveal melanoma with hepatic metastasis is extremely poor, and the overall survival is generally short: less than 1 year in most cases. Immunoembolization seems to be safe, easy to administer, and potentially effective. The results obtained in the study are encouraging; however, further clinical and basic research is needed to optimize and improve the efficacy of immuno- embolization.

### Galectin-9

Galectin-9, a β-galactoside-binding lectin, is closely associated with reduced metastasis and low recurrence in patients with malignant melanoma [31]. Intravenous galectin-9 administration reduced lung metastasis of B16F10 in an experimental mouse model [32]. It was reported that galectin-9 treatment could expand the population of unique macrophages with a plasmacytoid cell-like phenotype, and therefore promotes NK cell-mediated anti-tumor activity and significantly prolongs the survival of B16F10 melanoma-bearing mice [33].

### Homeopathy

In Europe homeopathy is very popular as a complementary and alternative medical therapy [34]. Many cancer patients treated with homeopathic approaches show an increase in their ability to fight cancer, improvement of their physical and emotional well-being, and alleviation of their pain resulting from the disease or conventional treatments [34]. Here, we describe the results of an experimental laboratory validation of the potential of peritoneal macrophages, challenged with a complex homeopathic medication, to stimulate the immune effectiveness of mesenteric lymph node lymphocytes. This new form of immunomodulatory therapy is based on Hahnemann's ancient homeopathic techniques, which use diluted substances that are vigorously shaken during preparation. Macrophages activated with a complex Brazilian homeopathic medication showed an improvement in the anti-cancer immune response against a very aggressive lineage of melanoma cells when co-cultured with lymphocytes [35].

### Microbe strategies

Vaccination with intracellular pathogens such as Bacille Calmette-Guerin (BCG), vaccinia virus, and *Chlamydophila pneumoniae* significantly decreased the incidence of melanoma [36] and increased survival in metastatic melanoma [37]. It was observed that microbes induce the cytotoxicity of macrophages toward melanoma, which is illustrated by the increased complex antitumor response such as expression of CCL2, CCL3, IL-6, CXCL10, CCL7, CD80, CXCL11, CXCL9 and IL-23 by macrophages [37].

### Nanoparticles

It was reported that polyhydroxylated fullerenols (Gd@C_82_(OH)_22_ nanoparticles) could induce murine melanoma cell death *in vitro*, and inhibit tumor formation and metastasis *in vivo*, by directly promoting macrophage viability, phagocytosis, and the secretion of cytokines by macrophages [38].

### Th1 cells

**Figure 3:**
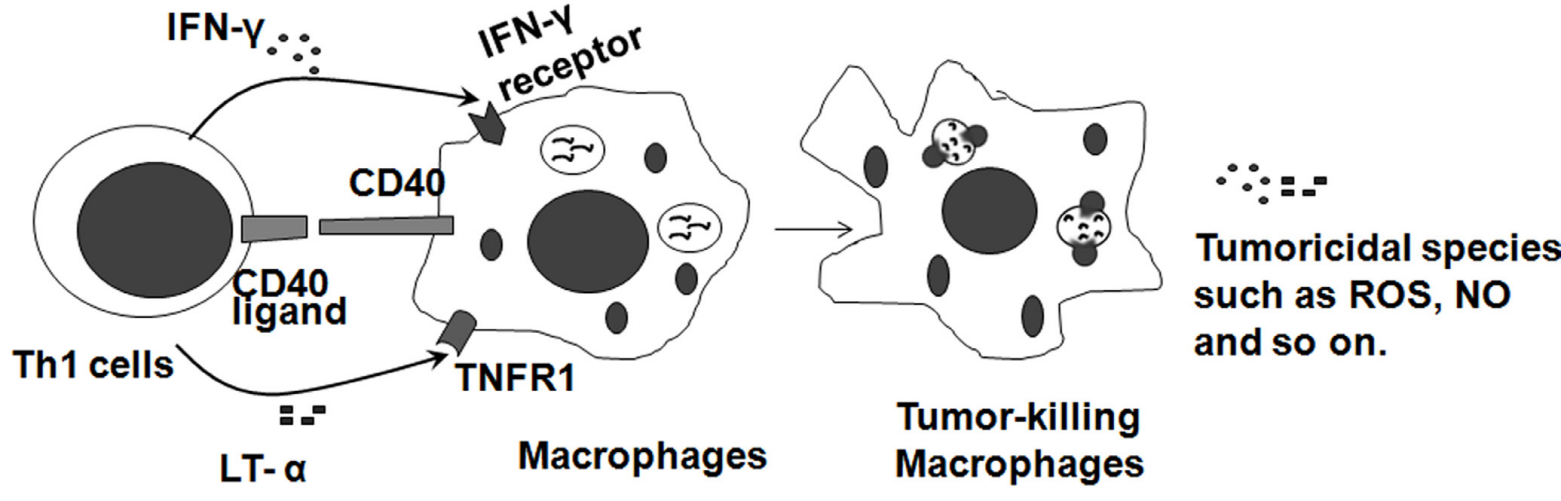
Tumoricidal macrophages are induced by Th1 cells Th1 cells characteristically express IFN-γ, CD40 ligand, lymphotoxin-alpha (LT-α) by their receptors on macrophages to stimulate macrophages into the potent tumoricidal effector cells, which generate tumoricidal reactive oxygen and nitrogen species, and enhance fusion of phagosomes with lysosomes.

Th1 cells have an important role in the tumoricidal activity of macrophages. Th1 cells produce cytokines to stimulate macrophages into the potent tumoricidal effector cells (Figure 3). In a Phase I clinical trial investigating the biologic activity of vaccination with irradiated autologous melanoma cells engineered to secrete human GM-CSF in patients with metastatic melanoma, it was demonstrated that immunization sites were intensely infiltrated with T lymphocytes and macrophages in all 21 evaluated patients and resulted in extensive tumor destruction [39]. In early reports, it was shown that Th1 cytokine mRNA for CD3δ, lymphotoxin (TNF-β), and IL-2 were significantly elevated in the ten regressing melanomas compared to the ten non-regressing melanomas [40]. IFN-γ mRNA was also elevated in regressing melanomas but failed to reach statistical significance [40]. This study shows an association between Th1 cytokines and spontaneously regressing melanomas. High expression of Th1-biasing cytokines IL-12 and IFN-α leads to a Th1-like phenotype and suppressed melanoma growth [41]. Another important function of Th1 cells is the recruitment of macrophages. Th1 cells produce the hematopoietic growth factors IL-3 and GM-CSF, which stimulate the production of new phagocytic cells in the bone marrow.

Vice versa, activated macrophages secrete IL-12, which increases the amount of IFN-γ produced by Th1 cells and also promotes the differentiation of activated naive CD4 T cells into Th1 effector cells. Interleukin-12-transfected B16 melanoma showed retarded tumor growth in syngeneic mice [42]. In a recent study, neoadjuvant local low-dose gamma irradiation can program the differentiation of iNOS M1 macrophages to exert anti-tumor effects on a xenotransplant mouse melanoma model through iNOS by inducing endothelial activation and the expression of Th1 chemokines and by suppressing the production of angiogenic, immunosuppressive, and tumor growth factors [43].

## BIPHASIC FACTORS

### IFN-γ

**Figure 4:**
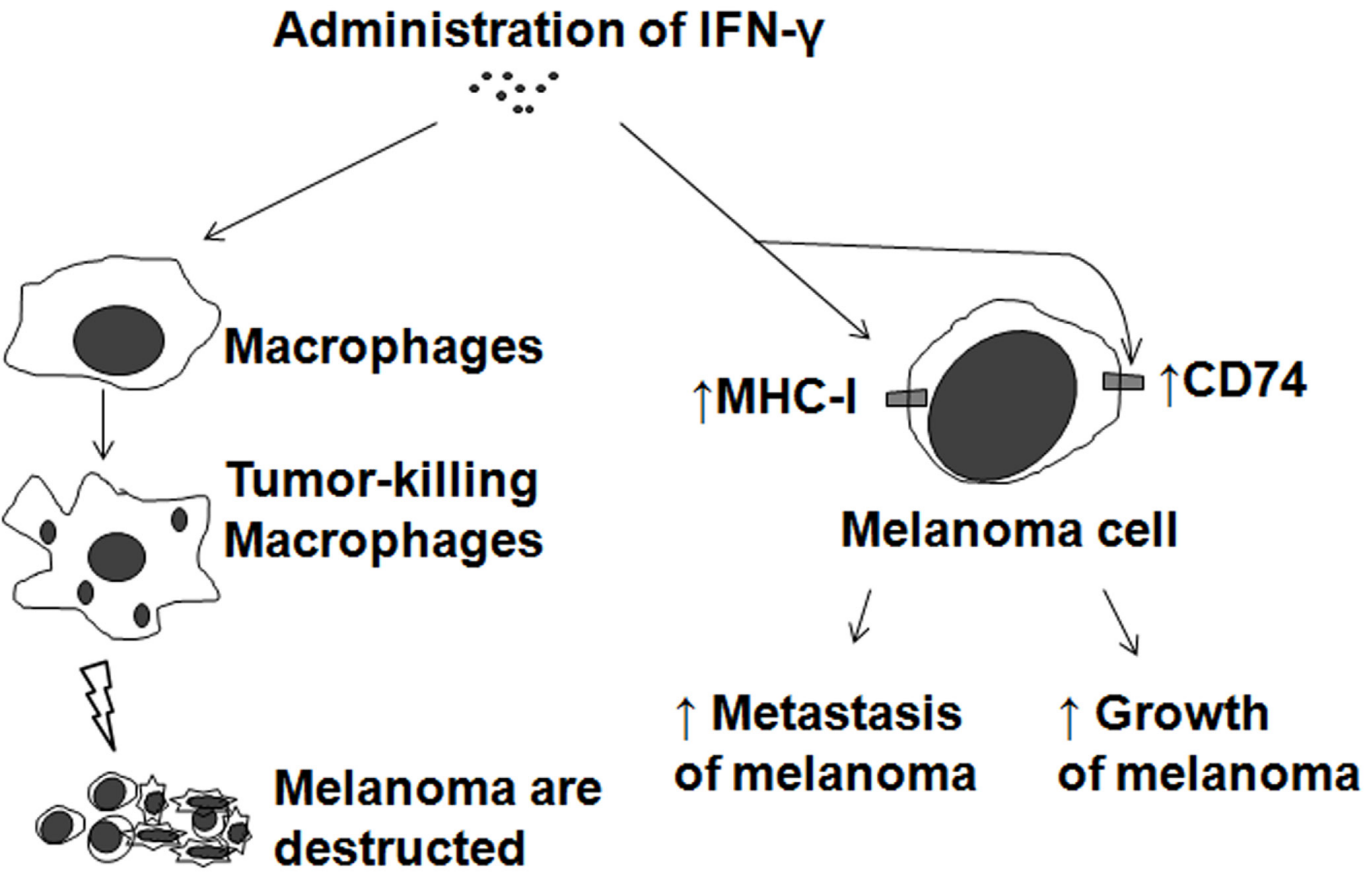
Biphasic effects of IFN-γ on melanoma Application of IFN-γ to melanoma treatment (Left). Adverse effects of IFN-γ on melanoma by pro-expression of MHC-I or CD74 (Right).

IFN-γ is the effector cytokine of Th1 helper cells and after Th1 activation macrophages, as the main effector cells of Th1 immunity, are activated to kill microorganisms and tumor cells and produce copious amounts of proinflammatory cytokines (Figure 3). Therefore, administration of IFN-γ suppresses melanoma development by activating macrophages (Figure 4 left). IFN-γ, which can be produced by macrophages, has a direct antitumor effect on melanoma cells [44–47]. IFN-γ plays a role in the response to melanoma indirectly through its effect on the immune system and directly through its anti-proliferative and pro-apoptotic effects on melanoma cells [44]. IFN-γ added to A375 melanoma cells caused an additive growth inhibitory response [45]. The combination of IFN-γ with IFN-α or IFN-β resulted in a strong synergistic anti-proliferative activity on four human melanoma cell lines (StML-11, StML-12, StML-14, and SKMel-28) [46]. The changes in gene expression associated with the direct anti-melanoma effect of IFN-γ were striking, as these involved genes or groups of genes previously implicated in the malignant phenotype of melanoma as well as genes not previously thought to be involved in melanoma growth and survival [47].

Several reports have suggested that IFN-γ may also have pro-tumorigenic effects in solid tumors under certain circumstances [46, 48]. Although IFN-γ reduces cellular growth *in vitro*, when introduced *in vivo* along with intravenously inoculated B16 melanoma cells, it induces lung colonization and enhanced expression of class I major histocompatibility complex antigens (Figure 4 right) [46, 48], which are more frequently expressed in advanced melanoma and related to an increased risk of metastasis in primary melanoma [46]. Elevated levels of IFN-γ may be an independent predictor of disease recurrence and may be used to identify a group of early-stage melanoma patients who are more likely to have recurrence of disease and who may benefit from adjuvant therapies, including immunotherapies [49]. In fact, a Southwest Oncology Group-randomized clinical trial showed that IFN-γ had an adverse effect on melanoma relapse and mortality rates [50]. In an ultraviolet B-irradiated mouse skin cancer model, macrophage-produced IFN-γ promotes melanoma growth by inhibiting apoptosis [51]. Pro-tumorigenic effects of IFN-γ may be, in part, due to pro-expression of CD74 in melanoma [52] (Figure 4 right).

### Monocyte chemoattractant protein-1 (MCP-1)

MCP-1 is a potent macrophage-recruiting molecule [53]. It was reported that MCP-1 was expressed during the early stages of human malignant melanoma, and it recruits macrophages and promotes tumor angiogenesis and growth [54]. However, MCP-1 action is biphasic in that high levels promote massive monocyte/ macrophage accumulation and tumor destruction, whereas low or intermediate levels support tumor growth [55].

### IL-1β

IL-1β is a pleiotropic pro-inflammatory cytokine involved in cell growth, differentiation, and regulation of immune responses [56]. It has been reported that the expression levels of the IL-1β gene or protein are associated with the invasiveness and metastasis of melanoma [57]. Metastatic melanoma cell lines do not secrete IL-1β but promote IL-1β production from macrophages [58]. A better understanding of the mechanisms and consequences of IL-1β production by infiltrating macrophages may be of interest for the development of IL-1β targeted therapy, such as anti-IL-1β antibody (canakinumab), against metastatic melanoma. On the other hand, IL-1β production from tumor cells may be considered a threat by the host's immune system. In this aspect, it has been reported that IL-1β-producing melanoma cells induce reduced tumor growth by recruiting immune cells [59].

## BLOCKAGE OF MELANOMA INHIBITION TO MACROPHAGE MIGRATION

**Figure 5:**
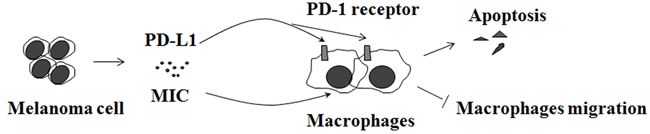
Melanoma inhibits macrophages Melanoma cell release programmed cell death-1 ligand 1 (PD-L1) and macrophage inhibitory cytokine-1 (MIC) that suppress the macrophage activation.

Melanoma inhibits macrophage activation by suppressing TLR-4 signaling [60]. Tumors may also express co-inhibitory or immune checkpoint proteins that shield them from attack by immune effector cells, such as macrophages [61]. For example, melanoma cells release programmed cell death-1 ligand [62], activation of its cognate receptor in macrophages [63], leads to apoptosis of the latter (Figure 5). It was observed that melanoma cell lines and metastatic melanomas expressed larger amounts of macrophage inhibitory cytokine-1 (MIC-1) than melanocytes, nevi, and primary lesions of melanoma [64–69]. Melanoma cells produce MIC-1 to protect against NK cell-mediated killing by inhibition of macrophage migration [69] (Figure 5). Knockdown of MIC-1 expression in melanomas resulted in a significant decrease in tumorigenicity [64, 70] by retarding vascular development [71]. CD74, also known as major histocompatibility complex class II-associated invariant chain, has been identified as the high-affinity receptor for the cytokine MIC-1 [72, 73]. It was reported that CD74 is expressed in melanoma but not in benign melanocytes using a melanoma progression tissue microarray [66]. In a xenograft melanoma model established by cell surface CD74-negative MeWo cells subcutaneously injected into the flank of SCID Beige mice, MIC-CD74 inhibition by MIC inhibitor ISO-1 suppresses tumor growth significantly [52].

## CHALLENGE OF PRO-TUMOR M2-TYPE MACROPHAGES

It should be noted that, owing to their plasticity, macrophages can be re-educated to adopt anti-inflammatory or pro-tumor M2-type, which often happens during late stage of inflammatory reaction. Studies using the B16 melanoma model have documented a gradual shift of initial Th0-, mixed Th1-/Th2-type CD4T cell response to Th2/Treg-type dominated responses by 14-20 days of progressive tumor growth [42, 74–76]. CD4Th2 cells and various regulatory cells produce cytokines such as TGF-β1 and IL-10 that can educate the macrophages to become protumor M2-type. Injection of neutralizing anti-IL-4, -IL-10, or -TGF-β1 antibodies can prevent this tumor-induced functional transition, resulting in enhanced CD8+ CTL generation and protection against tumor growth [74]. Depletion of CD4 T cells with locally secreted IL-12 in late-stage progressive B16 models, where Th2/Tr-type response dominate, eliminates Th2 cells and results in a Th1-dominant cytokine profile in tumor draining lymph nodes and leads to a retarded tumor growth in syngeneic mice [42].

Another solution to avoid a worst case scenario in the therapeutic strategy of macrophage activation is to use a high dose of macrophage recruitment factors, which can cause a massive accumulation of macrophages in a very short time. This artificial reaction is similar to the earlier stage of inflammatory reaction and the recruited macrophages are prone to fight the tumor, which is illustrated by application of MCP-1 [55] and GM-CSF [28].

## DISCUSSION

Macrophages serve as a first-line of defense against pathogens and environmental insults through release of anti-microbe mediators such as pro-inflammatory cytokines [77]. Cancer cells and their cellular debris do not present the types of proteins specific to the surface of healthy somatic cells, which stimulates macrophages to devour the cancer cells. Therefore, macrophages are important components of the innate immunity against tumors [9]. However, the complexity of tissue environments may render macrophages, which already possess functional diversification and plasticity, able to acquire pro- and anti-inflammatory properties, which then can be classified into two types, as pro-inflammatory or anti-tumor M1-type and anti-inflammatory or pro-tumor M2-type macrophages [78]. Moreover, a large body of evidence points to the tumorigenic populations of macrophages, and the macrophage-mediated anti-tumor therapy is faced with being abandoned.

Recently, we found that macrophages from D7Ertd443e knockout mice exhibit tumoricidal activities and adoptive transfer of both bone marrow-derived and peritoneal macrophages, after stimulation with M-CSF *in vitro*, results in inhibition of melanoma growth *in vivo* (data not published). Genetically modified cell-based vaccines encoding cytokines and co-stimulatory molecules allow sustained local release of cytokines to enhance a potent local inflammatory response without generating systemic side effects. GM-CSF appears to be the strongest promoter of local macrophages that can cause a macrophage-mediated anti-tumor inflammatory response at the vaccination site. It is expected that targeting the anti-tumor activity of macrophages is a promising approach for melanoma inhibition. We also suggest that the combination of tumoricidal macrophage activation and other treatments such as surgery, chemotherapy and radiotherapy, could be an effective and comprehensive anti-melanoma strategy.
